# Physical Activity: Exploring Views of Older Russian-Speaking Slavic Immigrants

**DOI:** 10.1155/2011/507829

**Published:** 2011-10-23

**Authors:** Janet Purath, Catherine Van Son, Cynthia F. Corbett

**Affiliations:** College of Nursing, Washington State University, P.O. Box 1495, Spokane, WA 99210, USA

## Abstract

Many of the 1.3 million Russian-speaking immigrants in the US have chronic conditions such as cardiovascular disease, diabetes, obesity, and depression. They engage in physical activity less often than other groups, and little is known about their views of physical activity. This qualitative study explored physical activity attitudes, beliefs, motivators, and barriers among older Russian-speaking immigrants. In four focus group interviews, 23 participants discussed physical activity. “Movement is life” was a theme throughout all interviews. Walking was the most frequently mentioned activity. Increased energy and decreased pain were described as health benefits. Motivators for physical activity were maintaining function, improved health, and the support of God and family. Barriers included poor health and environmental safety concerns. Participants suggested community walking groups and church-supported programs as useful methods to promote physical activity. Future research includes developing culturally appropriate interventions that utilize physical activity to prevent and manage chronic illness with ethnic minority older adults.

## 1. Introduction

Millions of Americans suffer from chronic conditions that can be prevented or improved through physical activity. Although the benefits of physical activity are many, most adults remain sedentary and are not active enough to improve their health [1]. Evidence suggests that the 1.3 million Russian-speaking Slavic immigrants (hereafter referred to as Slavic immigrants) living in the United States have even lower levels of physical activity than the general population. One study of Slavic women reported that nearly 70% had a sedentary lifestyle [2]. A survey of 176 adult Slavic immigrants reported that only 36.5% of participants engaged in moderate physical activity 5 or more days per week [3].

Slavic immigrants report multiple health problems with higher prevalence rates than their counterparts in the United States. Up to 90% of adult Slavic immigrants are overweight or obese [2–5]. Many are plagued with cardiovascular disease, diabetes, obesity, and depression [2–5]. Depending on the source, 64–90% report hypertension [2–6]. Depression rates are reported at 31% [3] and as high as 77% among women [5]. The high prevalence of chronic conditions among Slavic immigrants suggests that they might benefit from increased physical activity. Thus, intervention strategies to initiate and maintain physical activity in this population are urgently needed. Clarifying the relevant issues and unique physical activity challenges faced by Slavic immigrant older adults is an essential prerequisite for developing strategies to facilitate physical activity with this group.

Little is known about the physical activity beliefs of older Slavic immigrants. The views of other ethnicities are described by Belza and colleagues [7] who reported on research findings that examined barriers to and motivators for physical activity among seven different groups of under-served ethnically diverse older adults. Significant differences in barriers, motivators, and physical activity preferences were noted among each of the cultures studied. Slavic immigrants were not assessed in Belza and colleagues' research. In addition, men and women were grouped and gender differences were not evaluated. In order to develop interventions to increase physical activity for the Slavic immigrant population, culturally embedded health beliefs related to physical activity must be explored. The purpose of this study was to describe culturally embedded motivators and barriers to physical activity and to begin to identify culture- and gender-specific interventions to promote physical activity among older Slavic immigrants.

## 2. Materials and Methods

### 2.1. Design

A qualitative descriptive methodology was used, including focus groups and summative content analysis [8, 9]. Focus groups are a research method that has been shown to be effective in generating an understanding of beliefs and attitudes on a particular topic [10].

### 2.2. Sample

The four focus groups included men and women over 67 years of age. Participants were recruited by purposeful sampling and were conducted in two different geographical/metropolitan areas of Washington State to increase sample variation. There were 4 to 8 participants per group. Fifteen men and 9 women participated. Men and women were separated by gender because the population's conservative religious beliefs encourage women to defer to men in group conversation.

The groups' mean age was 72.9 years. Most participants had been in the US less than 10 years. All participants were over 50 years of age when they immigrated to the US with the average age at immigration being 65 years. The majority came from Ukraine (54%). Other countries of origin were Russia, Uzbekistan, Belarus, and Moldova. Seventeen per cent did not identify a country of origin. Nearly 60% of the participants were married. Average formal education was 8.1 years. All participants were Russian speaking, and none were fluent in English. Only 15 (62.5%) of the participants could walk 1/2 mile, and 22 (91.7%) reported that they could walk up a flight of stairs without difficulty.

### 2.3. Procedures

Washington State University and the Oregon Health and Science University's institutional research review boards approved the study, and written informed consent was provided by all participants. Study announcements were made within the Slavic community at a Slavic harvest festival, citizenship classes, and congregate housing sites. A Russian-speaking research assistant returned telephone calls and answered questions from potential participants. Each participant received a small honorarium.

Focus groups met at a place familiar to and convenient for the participants. Moderators were native Russian-speaking undergraduate and graduate nursing students who received training and mentoring from the investigators prior to the study. The Russian language was selected for the interviews because it was the language used in all schools in the former Soviet Union. Investigators took observational field notes related to body language, mobility, and group dynamics. In addition, investigators clarified questions and concerns from the moderators during the focus groups. Focus group prompts included questions related to motivators and barriers to physical activity [7]. To gain information about interventions that may be useful in promoting physical activity among older adult Slavic immigrants, two questions that were not included in Belza and colleagues research were asked to these participants: “What can healthcare providers do to assist you to start and/or maintain physical activity?” and “What can the community do to assist you to start and/or maintain physical activity?” Focus groups were conducted in Russian and lasted 45–60 minutes.

Focus group discussions were digitally recorded, transcribed by a professional transcription service, and then translated into English. At the end of each focus group demographics, information about the length of time since immigration, age at the time of immigration, and ability to walk 1/2 mile, and ability to use the stairs was obtained.

### 2.4. Analysis

All members of the research team independently coded the transcripts line by line and made observations noting insights and reflections of certified Russian-speaking translators which served as resources to clarify discussion context following translation when the researchers had questions about the group members' comments. The team then met to review and discuss their coding and to identify similarities and differences in themes. Common threads were considered further and refined via analysis until a particular descriptive theme was established by the data. Themes were shared with the focus group moderators to assess the credibility of the themes. Demographic characteristics were analyzed using descriptive statistics. To assure the participants' voice in the analysis, a member check with a subset of the older adult participants was conducted and compared to the investigator-identified themes.

## 3. Results and Discussion

### 3.1. Physical Activity Defined

Members of each of the four focus groups defined physical activity holistically as a part of living everyday life. Participants associated physical activity with all forms of physical movement with statements such as, “if one is moving, it means that he/she is alive”; “[one should be] an active participant in all events”. Physical activity was described as key to living, being able to care for one's self and others, and as being a gift from God.

“Movement is life…as you are moving, you are living. If one stops moving, the life is over, [it] is our life and health, and that is why it is important. God gives us this ability to move—so we would be able to take care of ourselves, to take care of and to serve others…there is no movement, death is inevitable”.

In all of the focus groups, walking was the single most frequently described physical activity and was seen as a method of transportation (as in running errands), as a form of leisure (as in walking with family members), as a way to maintain independence, and as a way to help others. Gardening was frequently discussed by participants and was seen as leisure, work, and as a mechanism for staying busy and moving around. Both genders preferred outdoor activities, “in the fresh air.”

“We need to walk, wiggle, take care of our gardens, and walk to the store, Russian store, there, and back, one hour there, and another one back, that's our (activity) program.” “We have our gardens…. We get up in the morning and go there, to the gardens where we dig, staying busy, moving around…. We are busy, we are working. We have activity.”

In listing specific examples of physical activity, gender differences became apparent. Men typically discussed leisure activities such as gardening, working in the yard, fishing and, less frequently, the use of exercise machines (e.g., bicycles and treadmills), running, and swimming. Fitness clubs were seen as prohibitively costly. Most men discussed walking and then various exercises that they did on their own at home.

Women, however, described doing home exercises, such as stretching and walking in addition to a long list of household activities. Household activities described included cookin cleaning, and laundry. “I am always busy doing something…kitchen, and work around the house in general.” A few women discussed activities such as going to work and being a caregiver for a parent.

### 3.2. Motivators

Overall, three categories of motivators emerged that were similar for both men and women in each of the focus groups: (1) participating in physical activity was desirable and seen as an acknowledgement of God's blessing of mobility and functionality, (2) fully engaging in family and social activities necessitated being physically active, and (3) maintaining health and independence was a benefit of physical activity.

#### 3.2.1. Relationship to God

Most participants believed that God predetermined their lifespan and that they were not able to change this. “Of course, by being a religious person, I understand that everything is in God's hands, no matter how hard I would exercise, I cannot add years to my life.” However, in all four focus groups, participants strongly emphasized that their relationship with God was key to their beliefs and behaviors towards physical activity. The belief that “God gives us the strength and…the ability to move” was frequently repeated. Faith was described as being interwoven daily with physical activity. This assisted them in the management of barriers related to the aging process and chronic conditions, motivating them to maintain current functional abilities.

“Thanks (to) God that we are still alive, that I am approaching 80 years. I am praising God for being able to walk on my own feet even though I am moving slow; that I am making everything with my two hands. This is why I am trying to stay very active, and trying to keep being active. As long as I can do it, I am doing it.”

#### 3.2.2. Family/Socialization

Relationships with family members and community also provided participants with motivation to engage in physical activity. Being physically mobile enabled them to interact with their families and be of service to others. Several participants noted that they engaged in physical activity because of grandchildren. “I am very inspired: I have twelve grandchildren, five great grandchildren. They all live in the United States and inspire me to do things. I want to walk because of them.” Another participant noted that to stay young, she needs to “do everything that children do…walk, run, play.” In addition to staying engaged with family members, staying active involved socializing with others.

#### 3.2.3. Health Management

Groups frequently discussed the aging process and the presence of chronic conditions. Participants described physical activity as helping them manage their aches and pains, enhancing sleep hygiene, reducing medication use, and promoting functional independence.

“Activity…suppresses the feeling of the existing pain. You overcome your pain and move, do things, and walk.” “…physical activity at our age is extremely important. As we keep moving, we are keeping our lives. Even though I have arthritis in all my joints, as I get up in the morning, first thing I do is light exercises.”

Thus, cyclical relationships among the motivators for participating in physical activity were described. Supported by their relationship with God, participating in physical activity enabled them to maintain function and promote health, leading to active engagement with family members and staying socially involved with their community, motivating them to continue to participate in physical activity.

### 3.3. Barriers

Barriers discussed were related to personal and environmental concerns and were similar for men and women. Physical concerns were first attributed to their advancing age noting that they were “worn out” and that they become “tired very fast”. However, these issues were soon further described to include specific chronic conditions or symptoms, such as pain, decreased stamina, fear of falling, and lack of desire constraining them from being as physically active as they would like to be.

Pain from conditions such as arthritis was seen as a barrier to participation in physical activity, especially walking. Decreased mobility and stamina often led to concerns regarding a fear of falling. “Physical activity can be…impossible to do. For instance, I have a very severe arthritis. As a result, I cannot walk for long periods of time. If I would try to walk longer distances, I would have to sit down for a while or I would start falling.” Fatigue was also identified as an issue. “I agree that it is important to go for walks. However, I am not always able…. I am tired on daily basis.”

Some participants made reference to struggling with lack of desire and how this influenced their ability to participate in physical activity. Other participants articulated similar thoughts.

“…all I want is to stay in bed…do not want to walk at all, no desire. But you have to pull yourself together and be active.” “You have to force yourself to do things…since our advancing age and sickness; weakness may interfere with our desires to stay active. But if one has the desire and the strength then that person can go for walks.”

Environmental barriers centered on safety, weather, and transportation. Safety issues included dogs running freely, heavy traffic, inadequate lighting in the evening, and fear of crime. One participant noted several of these environmental issues. She stated that there was not enough light on the streets, “for others the reason [they don't go for a walk] may be bad weather—too hot or too cold, or rain; some people… live in an area with lots of hills whereas others in the area with very heavy traffic”.

The presence of dogs running freely in the neighborhood stopped one participant from her evening walks. “I (used to) walk in the evening, but there are so many dogs on a street, they chase you…. I decided not to go for walks anymore.” Another participant shared how he was assaulted which keeps him from feeling safe walking in the evenings. Despite these barriers the general consensus from both men and women was that they needed to pursue being active, but sometimes doing so was difficult.

### 3.4. Bridging the Gap-Healthcare Provider and Community Support

When asked what they thought healthcare providers (HCP) could do for them, common responses for both men and women were, “They are already doing everything for us; do not know…. How much more could one take care of us?” Others claimed that their conditions were part of the aging process and not much could be done to influence this anymore. Participants valued communication with providers and they thought that the HCP should ask them about physical activity.

“…when a patient knows that someone cares about him and wants him to be healthy the patient will be more likely to carry on their exercise program…ask us: “how is your exercise program going?” We might need motivation. Doctors should give good advice. For example...what is the best physical activity for people of our age?”

When asked what the community could do to assist them to initiate or maintain physical activity several ideas were generated. Participants wanted education about different health conditions, benefits of physical activity, and how to promote their own health. Recommended teaching methods included: Russian language materials, visual aids, and demonstration. This conversation led to identifying the pros and cons of exercise classes, whether or not they would participate, and where classes should be held. Based on the initial themes, walking groups and church-based activities seemed to be viable ways of promoting physical activity. The investigators sought clarity about this during the member check, and both genders endorsed the preference for walking groups, church-based physical activity, and other health-promoting classes. 

The Slavic immigrant population is older than most ethnic minority groups in the United States [11], and chronic conditions are prevalent, amplifying the need to maintain health and deter morbidity. Addressing the unique motivators and barriers of this group requires culturally appropriate focused interventions.

As with other ethnicities, this older adult population identified walking as the exercise of choice [7, 12, 13]. Slavic older adults prefer low-cost or no-cost types of activity, as do other groups of older adults [7]. Similar to other researchers' findings, Slavic immigrant's physical activity is influenced by weather [14, 15] and personal safety concerns [16]. Health served as both a motivator and a barrier for physical activity, as with other previously studied groups [17, 18]. Because families are important, activity programs need to educate families on the importance of exercise for their older adult members and to provide them with information on ways they can facilitate their older family member's activity.

Unique to the Slavic older adults in this study was the influence of their Christian faith to motivate them to maintain mobility and participate in physical activity. Thus, acknowledging and embedding Christian precepts within the design of health promotion interventions and/or utilizing their faith-based community as a resource may serve to promote physical activity participation for this group of older adults.

Limitations to the study include the fact that participants espoused a strong Christian faith limiting generalizability to Slavic immigrants with other belief systems. In addition, this study included participants from at least five different and divergent areas in the former Soviet Union. The focus groups did not tease out unique views about physical activity barriers and motivators based on participants' area of origin.

Past research identifies that healthcare providers can play a key role in encouraging and supporting physical activity with the older adult population [18–21]. Specifically, when healthcare providers encourage physical activity, moderately disadvantaged and minority participants can overcome barriers to physical activity [19]. Although this group acknowledged barriers with healthcare providers, the relationship is valued and is a resource that should be utilized in the promotion of physical activity. An opportunity exists for healthcare providers to promote physical activity among Slavic older adults. With support and culturally appropriate materials, participants seemed to be open to exploring methods to enhance their current level of activity.

## 4. Conclusions

Russian-speaking older adult immigrants' descriptions provided a cultural lens for understanding views about physical activity not only as immigrants from the former Soviet Union but also as older adults. The focus groups addressed an important knowledge gap by exploring motivators and barriers to physical activity. Findings contribute to nursing and community health practice by: (1) bridging the knowledge gap about physical activity for this largely understudied immigrant group, (2) informing the design of the physical activity intervention for study of chronic illness prevention and management among Slavic immigrants, and (3) providing useful information for nurses, other health professionals, and community members working with Slavic populations.

Interventions should support leisure physical activity with a focus on increasing awareness of frequency and duration of activity as beneficial for health. With the older adult population, interventions will need to be adapted to address identified barriers. The goal of our research is to develop a culturally congruent chronic illness prevention and self-management program in which physical activity is a critical component. 

Knowledge of the differences in motivators for men and women will provide insight into the cognitive processes that relate to physical activity within this Slavic ethnic group. In addition, it is critical to identify these motivators within a cultural context as they can significantly influence the value of and participation in physical activity [22]. These insights are fundamental to understanding behavior change and developing interventions that support Slavic older adults′ unique cultural needs.
